# Notes on Two Cases of Urinary Calculus

**Published:** 1913-06

**Authors:** C. A. Moore

**Affiliations:** Assistant Surgeon, Bristol General Hospital.


### NOTES ON TWO CASES OF URINARY CALCULUS.

BY

C. A. Moore, M.S. Lond., Ch.M. Bristol, F.R.C.S.,

*Assistant Surgeon, Bristol General Hospital.*

---

The condition of calculus in the urinary passages is always an interesting one, and there are special points about both the cases described below.

**Case 1.**—F. P., male, age 38, admitted July 19th. Complains of lumbar pain and increased frequency of micturition. The previous history contained nothing of importance beyond the fact that the patient had been in hospital in India for malaria while he was serving in the army. The present illness began three months ago with frequency of micturition every two hours by day and about three times at night. Three weeks later patient began to suffer from pain across the loins behind, below the last rib on each side, much worse on the right side. The pain was a dull ache, not of a colicky nature, and did not pass down towards the groin or testicle. A month before admission micturition became painful, the pain being in the hypogastrium and along the urethra, especially severe towards the end of micturition. Three weeks before admission blood appeared in the urine for the first time. It came on suddenly, and at first the urine was deeply blood-stained—the urine being almost pure blood, according to the patient's account. It gradually cleared until it was only faintly stained, but generally a few drops of blood passed towards the end of micturition. Just before admission the blood ceased, but the pain and increased frequency continued. No "gravel" was ever noticed in the urine. On admission the patient was pale and rather thin, but looked fairly well. Nothing abnormal was revealed by examination of the chest. On examining the abdomen there was a markedly tender spot just internal to the left anterior superior iliac spine. The kidneys could not be palpated, but there was marked tenderness when pressure was made in the loins behind. The lumbar spine was held somewhat rigid on stooping, but no pain was elicited on "crowding" of the vertebræ. Cystoscopy and rectal examination both failed to reveal any abnormality. The urine was acid, and contained a good deal of pus, but no blood corpuscles, and examination for tubercle bacilli was negative. The temperature chart showed curious elevations of temperature. At intervals of roughly a week, between July 19th and September 2nd, the temperature rose to 102 to 104 deg., with a rigor on several occasions, but no urinary or other symptoms accompanied these elevations of temperature. Skiagrams were taken, and clearly showed a small shadow in a position corresponding to the pelvic part of the left ureter, but none elsewhere. A second skiagram taken a fortnight later confirmed the appearance seen in the first.

In view of the doubt as to the side affected, I decided, at Mr. Morton's suggestion, to explore both urinary tracts through a median laparotomy incision below the umbilicus. The right kidney was distinctly larger than usual, but felt perfectly normal otherwise, nor could anything resembling a calculus be felt along the course of the ureter. The left kidney was distinctly smaller than usual, perhaps three inches long or a little less, but in consistence it felt perfectly normal. The abdominal part of the ureter contained nothing, but in the course of the pelvic ureter, and just outside the bladder, a small, hard mass could be easily felt, corresponding in position to the shadow seen in the skiagrams. This appeared to confirm the diagnosis of left ureteral calculus. The mid-line incision was therefore closed, and an extraperitoneal ilio-inguinal incision made. The foreign body which had been felt proved to be a calculus in the ureter, immediately outside the bladder wall. The ureter was incised and the calculus extracted. The incision was so inaccessible that no attempt was made to close it, and the wound was stitched up with a drainage tube passing down to the wound in the ureter. The calculus was the size of a large cherry-stone, partially divided into two by a groove. It weighed about nine grains. Urine was discharged along the track of the drainage tube for nearly three weeks ; this then ceased, and the wound became soundly healed. After the operation the patient expressed himself as quite free from pain in the right loin and elsewhere, and was discharged looking and feeling quite well.

A few months later I saw the patient again. He was complaining of dull pain in the loins and some increased frequency of micturition. The wounds were soundly healed, and a skiagram showed no sign of any calculus. Examination of the urine, however, showed it to be swarming with bacillus coli, and a course of autogenous vaccines was given. This failed to entirely clear the urine of the organism, but caused considerable relief of his symptoms, although he still complained of some hypogastric pain when I last saw him some months ago.

The chief interest of this case centres round the difficulty of diagnosis. Although the chief pain was in the right loin, the only calculus discovered was low down in the left ureter.

Some years ago a theory, originated by Thornton and Guyon, and supported by other surgeons, was much in vogue, the so-called reno-renal reflex. By this was meant a reflex pain caused in a healthy kidney by the presence of a calculus in its fellow. The possibility of this was denied by Morris, who held that pain in a healthy kidney never occurred except as the accompaniment of more severe pain in its fellow. At first sight reno-renal reflex pain seems a convenient explanation of the pain in the right kidney in the case described above. The right kidney appeared healthy to the hand in the abdomen, nor did a skiagram show any sign of calculus in it. Moreover, the pain was apparently caused by the stone in the left ureter, since all symptoms temporarily subsided with the removal of the calculus from the opposite ureter. However, the recurrence of slighter symptoms on that side on the onset, later, of *B. coli* bacilluria, obscures the issue, since obviously the pain might have been due to a *B. coli* pyelitis. The possibility of a reno-renal reflex pain cannot be lightly put on one side, since it is a well-known fact that a stone in the upper urinary tract may cause a reno-vesical reflex, with hypogastric and perineal pain and dysuria, when the bladder itself is perfectly healthy.

The only way to clear up the diagnosis seemed to be to explore both tracts through an abdominal wound. Although the shadow of the ureteral calculus was quite definite in all the skiagrams, one knows from experience that there are fallacies—due especially to the presence of phleboliths in the pelvic veins—to be guarded against in the interpretations of such skiagrams.

The more or less regular attacks of pyrexia, sometimes accompanied by a rigor, were another puzzling feature of the case, and it was never cleared up as to whether they were really due to the old malarial infection or not. Inquiry from the patient since he left the hospital showed that they have not occurred since, so they were presumably due to the urinary condition, although the last occurred some days after the operation.

**Case 2.**—E. F., male, age 28, admitted September 2nd, 1912. Since fourteen years of age patient suffered from pain in the left loin and groin, for which a varicocele operation was performed when he was sixteen, but it gave no relief. When aged nineteen his left kidney was explored in the Gloucester Infirmary and a stone removed. His symptoms were entirely relieved, and for eight years he remained free from pain, but it began to return about twelve months ago, and had been increasing in severity for the last three months before admission.

The pain began in the left hypochondrium and passed down into the groin and testicle of the same side. It was made much worse by jolting, and came on in agonising attacks of colic, accompanied by vomiting. Frequency of micturition had never been increased, nor did the pain cause any strangury; the urine had often contained a white deposit, but never blood.

Except for some pallor, the man looked well. The chest was normal, and nothing unusual could be felt in the abdomen beyond some tenderness in the left loin, where there was a scar in the usual position for lumbar nephrotomy. The urine was of sp. gr. 1015, acid, contained urates and a trace of albumin, but no blood or pus. A skiagram clearly showed the shadow of a large stone in the left kidney, but none elsewhere. The patient informed me that he was told that he nearly died of shock and hemorrhage after the first operation. When told that the scarring left by the first operation would probably render a second more difficult, and therefore more dangerous, he expressed himself willing to take any risk necessary to relieve him from his miserable plight.

The kidney was cut down upon through the old scar. It was quite fixed, and could not be moved, much less delivered, on to the surface, the upper half being buried in dense scar-tissue. A large calculus could be clearly felt through the thinned cortex of the lower pole. An incision was made through this on to the stone, and an attempt made to extract it with forceps. This failed owing to the small opening, and the stone being wedged in the remaining kidney substance and scar-tissue. The opening was therefore made as large as possible, the stone grasped with the fingers, and torn out by force. Fairly smart hemorrhage followed from the torn cortex. It was too inaccessible to be controlled by sutures, but was easily checked by firm packing with gauze. The man lost a fair amount of blood, but his pulse never reached 130, and his condition caused no anxiety. Two days later the gauze was removed and the wound sutured, no hemorrhage ensuing. He left the hospital on September 26th, completely relieved of all his symptoms. The stone consisted of uric acid and weighed $2\frac{3}{4}$ ounces.

The interest in this case lies in the fact that it was apparently a case of recurrence of stone after a previous removal. Apparently it was not due to a piece having been left behind after the previous operation, since the patient was entirely free from any symptoms for eight years.

While recurrence of a stone in the bladder not uncommonly follows removal, even by lithotomy, it appears to be a very rare thing for a kidney-stone to recur. A careful search through all the standard works on the subject yields only one reference to the possibility of such an occurrence. Freyer reports that in sixty-six operations for stone in the kidney five of them were for recurrences, and three of the original operations were performed by himself.

In the somewhat analogous case of gall-stones, experience proves how uncommon it is for stones to accumulate in a gall-bladder which has been once emptied by operation, but the reason for this immunity is not easy to understand. Even granted that it is possible by special dieting or drugs to prevent the formation of calculi in the kidney, it is unlikely that in hospital cases, at any rate, any such prophylactic treatment is ever systematically undertaken. It is still less likely that a hospital patient, once rid of his symptoms by an operation, would ever submit to treatment designed to prevent a recurrence.

